# Kin-FOG: Automatic Simulated Freezing of Gait (FOG) Assessment System for Parkinson’s Disease

**DOI:** 10.3390/s19102416

**Published:** 2019-05-27

**Authors:** Sara Soltaninejad, Irene Cheng, Anup Basu

**Affiliations:** Department of Computing Science, University of Alberta, 32 Athabasca Hall, Edmonton, AB T6G 2E8, Canada; soltanin@ualberta.ca (S.S.); locheng@ualberta.ca (I.C.)

**Keywords:** parkinson’s disease (PD), freezing of gait (FOG), kinect, simple walking (SW), walking with turning (WWT), standing mode (ST), trajectory, angular displacement

## Abstract

Parkinson’s disease (PD) is one of the leading neurological disorders in the world with an increasing incidence rate for the elderly. Freezing of Gait (FOG) is one of the most incapacitating symptoms for PD especially in the later stages of the disease. FOG is a short absence or reduction of ability to walk for PD patients which can cause fall, reduction in patients’ quality of life, and even death. Existing FOG assessments by doctors are based on a patient’s diaries and experts’ manual video analysis which give subjective, inaccurate, and unreliable results. In the present research, an automatic FOG assessment system is designed for PD patients to provide objective information to neurologists about the FOG condition and the symptom’s characteristics. The proposed FOG assessment system uses an RGB-D sensor based on Microsoft Kinect V2 for capturing data for 5 healthy subjects who are trained to imitate the FOG phenomenon. The proposed FOG assessment system is called “Kin-FOG”. The analysis of foot joint trajectory of the motion captured by Kinect is used to find the FOG episodes. The evaluation of Kin-FOG is performed by two types of experiments, including: (1) simple walking (SW); and (2) walking with turning (WWT). Since the standing mode has features similar to a FOG episode, our Kin-FOG system proposes a method to distinguish between the FOG and standing episodes. Therefore, two general groups of experiments are conducted with standing state (WST) and without standing state (WOST). The gradient displacement of the angle between the foot and the ground is used as the feature for discriminating between FOG and standing modes. These experiments are conducted with different numbers of FOGs for getting reliable and general results. The Kin-FOG system reports the number of FOGs, their lengths, and the time slots when they occur. Experimental results demonstrate Kin-FOG has around 90% accuracy rate for FOG prediction in both experiments for different tasks (SW, WWT). The proposed Kin-FOG system can be used as a remote application at a patient’s home or a rehabilitation clinic for sending a neurologist the required FOG information. The reliability and generality of the proposed system will be evaluated for bigger data sets of actual PD subjects.

## 1. Introduction

Parkinson’s disease (PD) is one of the most common progressive neuro-degenerative diseases with a worldwide prevalence of 22 per 100,000 person-years for all age groups, and up to 529 per 100,000 person-years in older populations [1,2]. The major cause for PD is loss of the dopaminergic neurons in the part of the brain that is called the substantia nigra (SN) which is responsible for controlling the movement of different parts of the body. Therefore, the main symptoms of PD are related to motor disabilities including rigidity, bradykinesia, slowness, tremor, and freezing of gait (FOG). However, it has other non-motor symptoms such as sleep disorder, cognitive changes, mood disorders, and fatigue. Estimates show that 60.5% of PD patients experience at least one fall and 39% of them have recurrent falls which can cause fractures [3]. Falls and fractures can cause disabilities, significant impairment in the quality of life, and death with a 10.6% rate [2]. Fall can be because of different PD symptoms of which the major one is FOG.

FOG is a brief episode of absence of forward progression of the feet despite the intention to walk. Patients have the impression that their feet are glued to the ground [4] and they lose control over their gait. FOG is transient and lasts from a few seconds to up to 1–2 min [4,5]. This phenomenon happens mainly during gait initiation, turning, performing dual tasks or approaching narrow spaces [6,7].

Even though current medical drugs can successfully relieve most of the motor symptoms, FOG is one of the least responsive to medical treatments [5]. However, external cueing to induce an external auditory or visual stimulus are promising for tackling FOG and helps resume natural gait for patients. Currently, FOG assessment is done based on movement experiments in a lab environment, self-reported diaries from patients, manual video analysis by specialists and specific symptom questionnaires, namely FOG-questionnaire (FOG-Q) [8]. However, these strategies can provide biased information on a patient’s daily experiences because of limitations, such as the ones listed below:

- The set up for experiments is different from a real environment at home resulting in different FOG patterns compared to a real one.
- Even though FOG-Q can provide relevant indicators for the identification and characterization of FOG, it is based on the patients’ opinions which are subjective.
- Assessments are only done a few times a year. This is not suitable for FOG detection since we need continuous observations on patients.
- Self-assessment of FOG by PD patients is unreliable since most patients often experience memory loss and dementia [8].

On demand cueing is more efficient than continuous cueing in decreasing the duration of FOG episodes. Hence, automatic prediction of FOG is essential for generating the cueing only when a FOG event occurs [9]. FOG investigation is challenging because of its unpredictable and unreliable nature. The current treatments and cueing techniques are just temporary solutions for limiting a FOG event time. Moreover, there are many unknown aspects of the pathophysiology of FOG and its relation to PD. Furthermore, walking styles of PD patients differ across subjects (including diverse motor anomalies) [10]. Conventional gait assessment for patients is mainly conducted by a clinician visual inspection and observation which produce results that are subjective and rely on the observer’s experiences [11]. Thus, it is crucial to get the knowledge of the rehabilitation process by quantitative gait analysis, and understand to what extent does a patient recover from the disease [11]. Therefore, accurate and automatic FOG assessment for PD patients is not only crucial for providing neurologists the disease status and progress, but it is also essential for earlier and more efficient use of cueing techniques for tackling FOG. The proposed research in this field is mostly for FOG detection for PD patients. However, in this paper an automatic and accurate system is proposed which is necessary for neurologists to assess the FOG status of PD patients. The continuous assessment of FOG status for PD patients by neurologists using the propose Kin-FOG system can control the FOG situation which can eventually prevent unexpected falls for patients. Furthermore, the proposed system can be used remotely to send a patient’s information to neurologists which can save significant resources for both patients and doctors.

The rest of this paper is organized as follow: Section 2 covers literature review. Section 3 explains the data set that is used in this research. The proposed Kin-FOG assessment system is explained in Section 4. Experimental results and conclusion are discussed in Section 5.

## 2. Literature Review

In the past decade, smart sensors, especially wearable ones have increasingly become a tool for assessment of motor symptoms such as FOG in PD and other movement disorders. This is because of improvements in computational power of small devices [12]. The proposed methods for FOG detection using these sensors are categorized into two groups depending on the type of the signal and the analysis method used. The first group uses electrical signals and the second group is based on gait information [13]. The first group assesses FOG by monitoring the physiological changes of signals, such as electroencephalography (EEG) [14] and electromyography (EMG) [15]. Despite a large number of studies that investigated the use of wearable sensors to detect gait disturbances, such as FOG and falls, there is little agreement regarding the most effective system design, e.g., types of sensors, number of sensors, location of the sensors on the body, and signal processing algorithms [6]. In [14], it is shown that during FOG episodes, the total amount of EMG activity is reduced in the lower limb muscles. The detection and monitoring of FOG with gait information including kinematic and kinetics is more convenient and easier [13]. The proposed methods for FOG detection is based on shallow machine learning algorithms which are applied to the signals acquired from the sensors. The researchers aim to extract some features which can distinguish FOG episodes from normal gait. As the first attempt in this field, Han et al. [16], explored FOG episodes and movement abnormalities in PD patients and other movement disorders by using an Unconstrained Activity Monitoring System (U-AMS). The proposed method used wavelets to discriminate between FOG and normal walking. FFT and amplitude analysis were used as the features to classify between FOG and non-FOG regions. The data set consisted of 5 PD and 2 normal subjects. The authors reported that the frequency response range for the 2 patients with accelerometers at the ankle was between 6 and 8 Hz. Moore et al. [17], presented a device for ambulatory monitoring of FOG using the frequency characteristics of vertical leg movement. They defined the freeze index (FI) as the power of the considered body segment acceleration signal in the “freeze” band (3–8 Hz) divided by the power of the signal in the “locomotor” band (0.5–3 Hz). A FOG event was detected when FI exceeded a certain threshold [18]. The experimental results were subject-dependent, and showed 78% correct detection of FOG (true positive rate) and 20% false positive rate.

The proposed methods for FOG detection can be real-time or offline. The first category is suitable for real-time applications. In 2009, Bachlin et al. [19] presented the MBFA method, which is a real-time FOG detection method, to fix the latency limitation of the method by Moore et al. [17]. They introduced a new term, Power Index (PI), defined as the addition of the Walking Band (WB) and the Freezing Band (FB) to indicate the amount of movement. In this paper, there are two thresholds which are Freezing Threshold (FTH) and Power Threshold (PTH). FOG episodes are determined if *FI* > *FTH* and *PI* > *PTH*. This FOG detection method has low computational cost and good performance. Furthermore, once the FOG episodes are detected the person gets auditory signals until resuming walking. The limitation of this work is the low number of patients. They reported 73.1% and 81.6% for sensitivity and specificity, respectively. During this time, Daphnet data set was created by the same authors for evaluation of FOG detection methods. However, the conditions defining the data collection protocol, such as the limited set of activities and the clinical settings, may overestimate the results of the approaches tested on it compared to performances obtained using real data. In 2010, Maidan et al. [20] tested the hypothesis that heart rate (HR) increases during FOG and just before FOG is going to happen. To evaluate these hypotheses, HR and HR variability were considered to be the features for the subjects who carried out tasks that frequently provoke FOG. The data set in this paper has 15 healthy older adults, 10 patients with PD who experienced FOG, and 10 patients who did not. Moreover, their results suggest that action observation has a positive additional effect on recovery of walking ability in PD patients with FOG. Delval et al. [21], proposed a FOG detection system based on gait analysis. The data for this paper was gathered by walking a group of subjects (10 PD with FOG, 10 PD without FOG and 10 Control) on a motorized treadmill while avoiding unexpectedly appearing obstacles. Treadmill walking was videotaped, and FOG episodes were identified by two independent experts. Gait was also analyzed using detailed kinematics properties. Knee joint signals were processed using time frequency analysis with combinations of sliding fast Fourier transform and wavelet transform. This approach using the time frequency features detected even very brief FOG with acceptable sensitivity (75–83%) and specificity (>95%).

Mazilu et al. [22] presented a novel FOG monitoring system based on the work of Bachlin et al. [19]. They used a smart phone and a wrist acceleration sensor for capturing the motion data. This paper was the first to use machine learning for detection of FOG. The features for doing this classification were mean, standard deviation, entropy, energy, FI, and power of the acceleration signal. The ML algorithms used were Random Forest (RF), Naive Bayes (NB) and K-nearest Neighbor (KNN). The best result obtained was 66.25 and 95.83 for sensitivity and specificity with RF. In the next year, they presented another automatic FOG detection system using wearable sensors. The feature learning and unsupervised methods were compared by time domain and statistical features from the motion data. The latter one has better performance by up to 8.1% in terms of F1-measure. The authors performed multi-class analysis since the pre-FOG is considered to be the new class (FOG vs. pre-FOG vs. normal locomotion). They also used auditory cueing at the end of their analysis for warning the patient about FOG episodes. Another real-time FOG detection method is presented by Zhao et al. [23]. An accelerometer integrated pant (MiMed-Pant) is used for capturing the gait information which sends the information to a wireless computer. The FOG detection algorithm is based on the frequency and time analysis and Power Spectral Density (PSD) features. The proposed method is executed every 0.5 s and the results are shown within the same time period. This is one of the positive aspects of this method. Evaluation is performed over 8 PD subjects in which the real FOG is determined for them by a specialist using recorded video. The limitation of this work is that the patient needs to wear the cumbersome MiMed-Pant for using the technique.

Electroencephalography (EEG) signals are used in the paper presented by Ardi [24] for FOG detection. The EEG signals are obtained using a 4 channel wireless EEG system developed for 26 PD patient at the Parkinson’s Disease Research Clinic at the Brain and Mind Research Institute, in University of Sydney. Preprocessing is done for the motion data by applying a high-pass and a low-pass filter for removing noise and unnecessary information. In this research, a discrete wavelet transform (DWT) based on dyadic scales and positions is used for feature extraction. In the classification phase, a three-layer Back Propagation Neural Network (BP-NN) is used, with 56% of the data used for training, 25% for validation, and 19% for testing. Experimental results show 0.75% accuracy for the proposed FOG detection system. In 2013, Moore et al. [25], proposed assessing different numbers of sensors in different locations for gait analysis with the purpose of FOG detection. The MBFA algorithm was used as the FOG detection algorithm with different sensor configurations. The data is captured by 7 sensors for 25 PD patients. Their analysis shows that by using all the sensor maximum accuracy is achieved for FOG detection which has high sensitivity (86.2%) and specificity (82.4%) when considered to be a binary test for the presence or absence of FOG.

In 2015, Zack et al. [26], presented a method for FOG detection using a triaxial linear waist-mounted accelerometer. Experiments for their paper include walking rapidly with short steps and rapid full turns in both directions, conducted on 23 patients. Two independent experts identified FOG episodes using offline video analysis (gold standard). In the proposed method FI is used as the discriminator, but a general threshold instead of a numerical one is applied to find the FOG episodes. Receiver Operating Characteristic (ROC) curves were drawn to determine a global FI threshold to distinguish between FOG and non-FOG episodes within the different tasks and for all tasks together. In addition to the global FI threshold, they calculated the sensitivity and specificity of the FI threshold for each subject. Combining all tasks together, sensitivity of 75% and specificity of 76% was obtained.

Tay et al. [27] presented a real-time PD monitoring and biofeedback system for FOG using low-cost wearable sensors (3 for each sensor on the neck, right ankle, and left ankle). Their proposed method detected FOG based on kinematic gait analysis using the gait cycle time and frequency features. The data set consisted of 8 PD subjects, 5 of whom have FOG episodes. Moreover, this system benefits PD patients from the periodic cueing to pace their steps after a FOG occurrence; hence, improving their gait performance. The system includes local storage capability which is useful for FOG detection when patients are outside their home or a clinic. However, no quantitative results were given.

In 2017, Rodríguez et al. [8] presented a novel approach for FOG detection using machine learning techniques based on daily activities of PD patients in a real environment. This paper has the advantage that the data is captured in a real environment. They extracted 55 FOG-related features from 21 PD patients using just one waist-worn triaxial accelerometer. SVM with leave-one-out cross validation was used for classification. Evaluation was done in two cases, including general (user-independent) and specific (user-dependent). Experimental results show significant improvement in accuracy of the personalized model compared to the generic model; with enhancement in the geometric mean (GM) by 7.2%. Following this work, Sama et al. [28] reduced the number of extracted features to 28 for the same data set. They also evaluated the extracted features by using 8 types of classifiers with greedy subset selection process, 10-fold cross validation, and different window sizes for signal analysis. The results show that the proposed method detects FOGs at a patients’ home with 91.7% sensitivity and 87.4% specificity, enhancing the results of former methods by 5% to 11% and providing a more balanced rate of true positives and true negatives.

Most of the papers presented use wearable sensors for collection of motion data because of their application in real life environments. However, using wearable sensors in an out-of-lab environment requires users to put the sensors in the correct positions. Such flexibility can cause variations in data capture which impacts the gait assessment quality. This is one of the major challenges for self-administration of wearable sensors by users in an out-of-lab environment without any supervision [29]. In [29], a framework is proposed for quantifying the variations resulting from using wearable sensors for data capture in a free-living environment. Even though there are many factors that affect data capture by wearable sensors, they consider the four more important sources of variations including mounting location, mounting leg, sensors, and speed. They statistically model this problem by using just one healthy non-elderly subject with a full-factorial design of 48 factors combinations. In [29] the influence of these four factors and their interaction on gait features derived by wearable sensors are characterized. As a result of their analysis, mounting location and gait speed were demonstrated as the most dominant sources of variation. There are many studies that use non-wearable sensors, such as Kinect as an assessment tool for gait analysis for PD patients. In [30], Kinect was used for body movement monitoring for PD patients with machine learning techniques. The proposed method can monitor gait abnormalities for PD patients with high accuracy not only for diagnosis but also for disease severity detection. To the best of our knowledge, there is only one paper [31] that used Microsoft Kinect for FOG detection. In this paper, a novel system is designed based on gait analysis of human motion to detect FOG and falling for PD patients. The novelty of this method is in monitoring and improving the mobility using laser-based visual cues, which is called Kinect4FOG. An algorithm was developed to monitor the behavior of a subjects’ gait cycle and the number of footsteps within a given time interval to estimate the occurrence of a FOG. A foot-off event is considered to have occurred when the knee angle of one foot has decreased to less than a specific threshold. The authors evaluated their method using over 15 PD subjects with FOGs who performed motion experiments using Microsoft Kinect V2. There was positive feedback on the proposed system in domestic usability, but it had limitations in outdoor use. This paper reported that 86.6% were satisfied with the FOG detection system whereas 13.3% neither agreed nor disagreed. The reported results were based on people’s feedback which are subjective. However, no objective results are reported.

Feature learning methods have also been used for automatic FOG determination. Among these, the most popular one these days is deep learning (DL). DL models learn feature extractions that can easily handle multimodal data, missing information, and high-dimensional feature spaces. Just a few papers are proposed for FOG detection using DL including [12,32]. Julia et al. [32] proposed a DL model which uses a 6-layer CNN network for FOG detection. The input for this network is the stack of compressed form of the current signal window at time *t* and time *t* − 1 which consists of 9 gyroscopes and acceleration features. The current method is better than all the state-of-the-art methods for FOG detection, achieving performances of about 90% for GM. However, DL-based methods need more computational resources than others in real-time applications for FOG detection. Also, DL needs a dataset that includes a large and representative set of examples to successfully train the network and obtain meaningful results.

Qiu et al. [11] presented a low-cost, intelligent, and light-weight platform for providing quantitative gait assessment information of stroke patients. They used multi-sensor fusion using accelerometer, gyroscope and magnetometer (MARG) for taking advantage of the individual sensors and avoiding their weaknesses. They reported different gait parameters for stroke patients such as step length, number of steps, and other gait cycle parameters. Moreover, they provided qualitative results which have useful information for the specialists about the situation of their patients. Another other gait assessment system was proposed for equestrian sport evaluation by Wange et al. [33]. This paper, similar to Qiu [11], used fusion of sensors as MARG for the rider on 7 parts of their body but no sensor was placed on the horse. They also used Northern Digital (NDI) Camera for capturing videos of the riders for evaluating their system. Pelvis joint was selected as the reference for gait analysis to distinguish between professional and novice riders. Until now, most of the proposed methods for FOG detection tries to warn patients before FOG happens. However, neurologists for assessing PD patients need to have an automatic, accurate, and fast system for getting the FOG information of the patients. In addition, most of the proposed FOG detection methods must tape a video while they are capturing the data and then do manual video analysis for generating the ground truth labels to evaluate their algorithm. We propose an automatic, accurate and fast FOG assessment system (Kin-FOG) which addresses these limitations by introducing the improvements listed below:

- The proposed Kin-FOG system uses an RGB sensor based on Microsoft Kinect which is more convenient than wearable sensors for elderly people. Moreover, it can capture video and motion data in parallel.
- The proposed system can present a patient’s FOG conditions to specialists for disease stage determination and treatment. The FOG status includes number of FOGs, length of each FOG, and the time when a FOG happened. The continuous assessment of patients by doctors with proper treatment can control FOG and eventually prevent patients from unexpected falls.
- The novel FOG algorithm can be used remotely by PD patients, at their home or other places such as senior homes, to send their FOG status to their doctors instead of going to a clinic. This will save significant time, money, and energy for patients and specialists.
- The proposed FOG detection technique can be applied on video captured by devices other than Kinect after extracting a subject’s pose from a video.

Gait assessment tools in clinics may have limited applications because of three main reasons including cost, complexity and specific space requirements [11]. Our proposed Kin-FOG system is cheap, user friendly with easy and fast set up. However, our FOG assessment system can only be used in a lab, clinic, or home environment, but not outdoor.

## 3. Proposed Method

**Figure 1:**
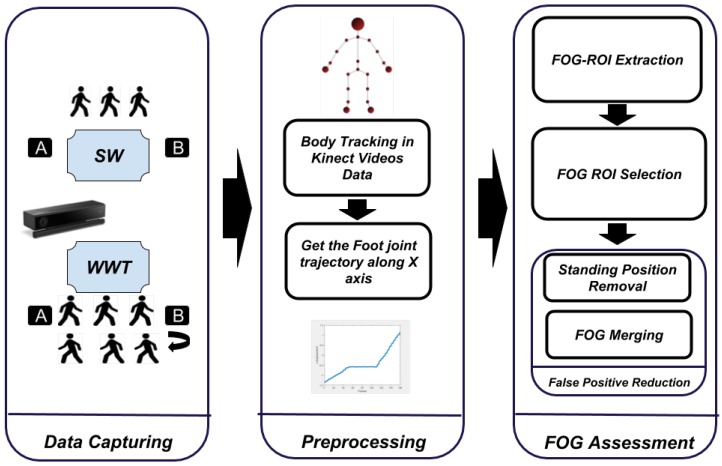
Kin-FOG framework for FOG assessment for PD patients.

In this paper, an automatic FOG assessment system is proposed for helping specialists with automatic assessment of FOG for PD patients and provide efficient and on time treatment. The general framework of the proposed system is shown in Figure 1. It has three phases, including data capture by Kinect, data preprocessing, and FOG assessment. All the steps of the Kin-FOG system are explained in the next subsections.

### 3.1. Data Acquisition

**Figure 2:**
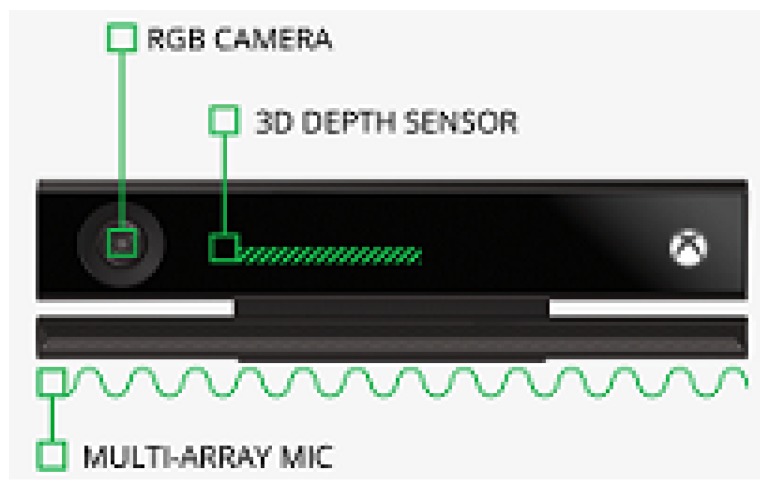
The components of Microsoft Kinect V2.

**Figure 3:**
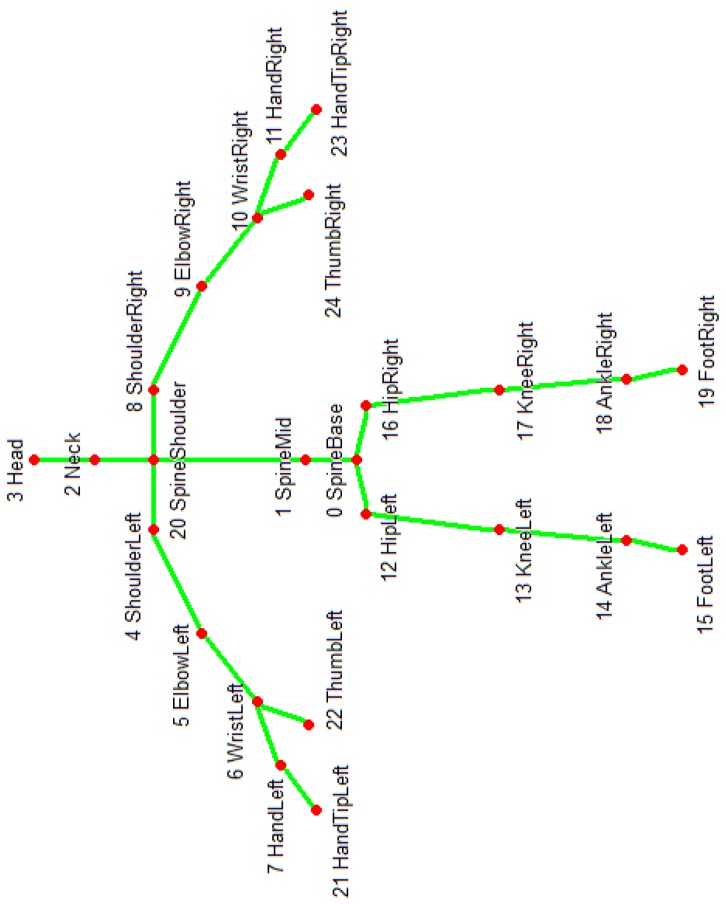
Kinect skeleton body joints with their indices. Kinect V2 can record motion data up to 25 joints.

The Microsoft Kinect V2 is a non-wearable sensor for capturing the 3D motion of a person. It was invented for Xbox console game devices which allow interactions with body movements, voice, and images. It has three main parts consisting of a 3D depth sensor, an RGB camera and multi-array microphones which are shown in Figure 2. Kinect V2 is cheap compared to other video capture devices and other motion capture sensors. It employs an RGB camera with a resolution of 1920 × 1080 at 25 fps and a depth sensor with an output resolution of 512 × 424 based on time of flight. The depth range is 0.5 to 4.5 m. Kinect V2 can detect up to 6 people, with each person represented by 25 joints. Figure 3 illustrates these joints of the body.

**Figure 4:**
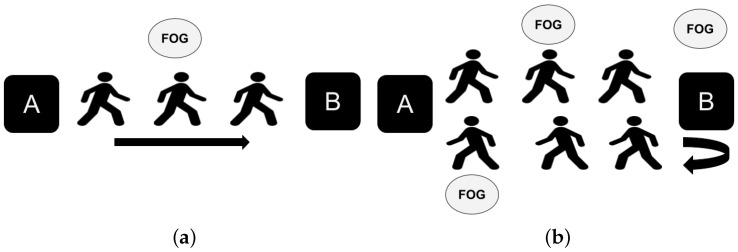
Different motion capture tasks in our experiments: (**a**) SW, (**b**) WWT.

In our study, Kinect is used to capture data from 5 subjects. The subjects are healthy without any movement disability. The data set consists of 1 female and 4 males. The average age of the subjects is 30.8. The subjects have different height and speed which is useful for having different types of data. Although the subjects are healthy, they are trained with real video on how FOG occurs for PD patients. The general information about the subjects is presented in Table 1. Two categories of experiments are done. The first experiment is in the without standing mode (WOST) and the second one is with standing (WST). In both categories two types of tasks are used which are described as below:

- Simple Walking (SW):
  The subjects are asked to walk from a point (*A*) to another point (*B*). They do this experiment with two different number of FOGs (1 and 2) between the start and stop points. Figure 4a shows the SW experiment scenario.
- Walking with Turning (WWT):
  The subjects are asked to walk from a point (*A*) to another point (*B*) and then turn and return to the starting point (*A*). They do this experiment with three different numbers of FOGs (3, 4, and 5). Figure 4b shows the WWT experiment. One of the FOG happens close to turning, and the rest occurs randomly between *A* to *B*.

The Kinect sensor is set on a table with height 47 inches. The distance between points *A* and *B* is 104 inches and the subject walks in front of Kinect at a distance of 97 inches. Please note that Kinect can cover a longer range but because of the space limitation in our lab we only consider this set up. The video with motion data is captured for each subject with SW and WWT tasks for WOST and WST modes.

The number of FOGs in these experiments are presented in Table 2. To design a general and reliable system different numbers of FOGs are conducted in these experiments. Each subject is asked to repeat the experiments 3 times and the best experiment with the highest similarity to a real FOG phenomenon is selected to make sure the data is captured correctly.

### 3.2. Preprocessing

In this phase, the data captured by Kinect is processed to get the body joint’s gait information and the proper motion signal for FOG assessment. In addition, the ground truth labels are extracted by checking the recorded video frames for final evaluation of the proposed method. The ground truth labels have three values including FOG, non-FOG, and non-related. The non-related labels are for the frames which are not related to the actual experiments.

#### 3.2.1. Body Skeleton Tracking

**Figure 5:**
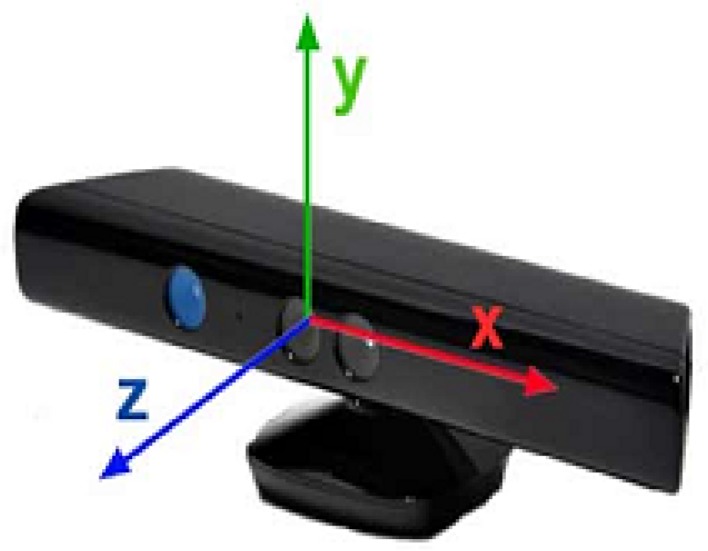
The coordinate system of the Microsoft Kinect V2 sensor.

The body skeleton and all the joints are extracted from the video data that are recorded by Kinect. Since Kinect records depth data, for each joint we have a list of 3*D* positions (*X*,*Y*,*Z*) and the length of the list is equal to the number of frames for the video. Figure 5 shows the coordinate system of Kinect when it is tracking the body joints. The *Z* axis data is the distance between the subject and the Kinect, which is almost stable in our experiments and thus provides no useful information. On the other hand, since the subject is walking along the *X* axis of the Kinect, the *X* trajectory data is selected for our FOG gait analysis.

#### 3.2.2. Joint Trajectory Selection

As mentioned before, FOG happens when a person’s foot is frozen and not able to move forward. Ahmadi et al. [34] proposed a low-cost system for human gait assessment which performs the following tasks: lower limb position estimation, orientation estimation, and 3D reconstruction of the estimated positions and orientations. They also propose a method for kinematic model adjustment at the end to cover the error from the wearable sensor’s position uncertainty. The position estimation is done just by using the captured motion of Inertial Measurement Units (IMU) sensors on the feet. For orientation estimation and 3D reconstruction they used ankle and knee joints trajectories as well. FOG is mostly related to the leg joints which are hip, knee, ankle, and foot. Since, most of the papers in this field use sensors which are attached to these joints, we decided to select the foot and ankle as our FOG-related joints. The foot joints (left and right) are joint numbers 15 and 19 and the ankle joints (left and right) are joint numbers 14 and 18 as shown in Figure 3. The *X* trajectory of the right and left foot for all the subjects are collected for FOG assessment in the proposed system.

**Figure 6:**
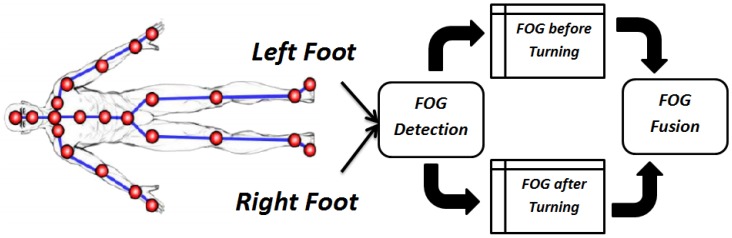
FOG assessment for the WWT experiment by our proposed Kin-FOG system using right and left feet. The left foot is used for ‘before turning’ and the right foot is used for ‘after turning’.

The SW experiment FOG assessment just needs the left foot *X* trajectory. However, the WWT experiment needs both left and right feet *X* trajectories to detect all the FOGs. In the WWT experiment the trajectory signal has two parts consisting of before turning and after turning. The left foot trajectory is used before turning and the right foot trajectory follows the same procedure for after turning. At the end, the analysis results of the left and right feet are fused to get the final FOG assessment for the WWT experiment. Figure 6 illustrates the process for the WWT experiment.

### 3.3. FOG Assessment

After getting the *X* trajectory of the right and left feet, the goal is detecting the FOG episodes based on these signals. The Kin-FOG system follows three steps for FOG detection and monitoring which are described in the next sections.

#### 3.3.1. FOG-ROI Extraction

**Figure 7:**
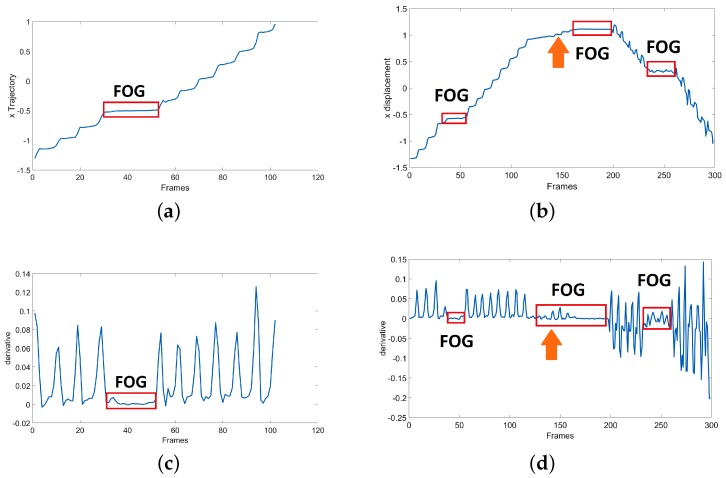
The *X* trajectory signals for: (**a**) SW with 1 FOG, (**b**) WWT with 3 FOGs. The turning point is marked with an arrow, and the derivative signals are: (**c**) SW with 1 FOG, (**d**) WWT with 3 FOGS.

As explained before, FOG episodes are the times when subjects are not able to move forward. Hence, FOG episodes are likely to be flat regions in the *X* trajectory of the foot joint. Figure 7 shows two samples of the *X* trajectory for the left foot of a subject for SW and WWT experiment tasks, in which the FOG regions are marked. Please note that the *x*-axis of the plots are frame IDs and the *y*-axis shows the *X* displacements. In Figure 7a the trajectory signal is related to the SW experiment with 1 FOG. The Figure 7b shows the *X* trajectory of the same subject in the WWT experiment with 3 FOGs which happen one before turning, and two after turning. The Turning Phase is marked with the orange array in this figure. Please note that the turning point is computed by finding the maximum point in the *X* trajectory plot. The *X* trajectory is defined as *f*(*x*) for simplicity in clarifying the mathematics behind the proposed Kin-FOG assessment system.

For extracting the flat regions in the signal, we must compute the derivative and then find the regions equal or close to zero. Qiu et al. [11] used similar techniques on foot joint trajectory for stance phase detection in the gait cycle. Figure 7c,d illustrate the derivation of the previous *X* trajectory signals. The first plot, Figure 7c, is related to the SW experiment and the second plot, Figure 7d, shows the derivation signal of the WWT experiment. As shown, FOG episodes have derivatives close to zero. All these regions are going to be extracted as FOG region of interests (ROIs) and called FOG-ROIs. The derivative of the trajectory signal *f*(*x*) is computed based on Equation (1).

(1) $$g\left(x\right)=f^{{}^{\prime }}\left(x_{i}\right)=\frac{f\left(x_{i}\right)-f\left(x_{i-1}\right)}{f_{i}-f_{i-1}}\;i=\left(1:n\right)$$

where *g*(*x*) shows the derivative of the trajectory signal. The gradient computed by finding the distance between the displacement value of frame *i*, *f*(*x_i_*) and the displacement value of the previous frame *f*(*x_i_*_−1_). Also, *n* is the total number of frames. We find all the areas with derivative close to zero based on Equation (2). These regions are the candidates of FOG.

(2) $$h\left(x\right)=\left\{\begin{array}{c} 1,\mathrm{if}|g(x)|\leq \epsilon \\ 0,\mathrm{otherwise} \end{array}\right.$$

#### 3.3.2. FOG-ROI Selection

In the previous step, all the FOG-ROIs are extracted from the trajectory signal as candidates for FOG episodes. However, not all of them are real FOG episodes. For example, sometimes the derivative in just one frame is zero and the adjacent frames are not. This cannot be FOG since it is very short.

**Figure 8:**
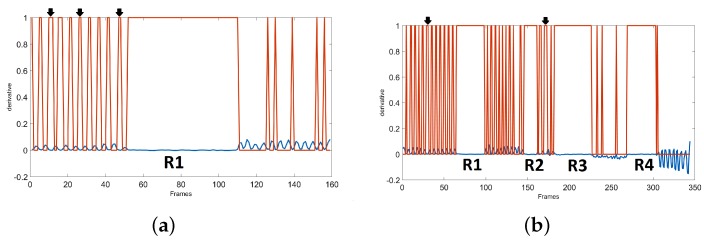
The derivative plot in blue with the *h*(*x*) plot in red. Some samples of non-FOG regions with zero derivative are shown with an arrow. R1, R2, R3, and R4 are the real FOG regions. (**a**) SW with 1 FOG, (**b**) WWT with 4 FOGS.

Figure 8 plots the *h*(*x*) regions which is 1 for the area with derivative close to zero and is 0 for other areas. It can be seen that most of these regions are not related to FOG. Some samples of these non-FOG regions are shown with an arrow in Figure 8 for SW and WWT signals from the previous sections. Therefore, we must check *h*(*x*) and find the places where there is a group of continuous 1 s which has “*uniform*” FOG patterns. The non-zero areas are found, and their lengths are computed. Then, in our Kin-FOG system the areas with length higher than a threshold (*FOGLen_Thr_*) are selected as real FOG regions. By following this process, we ignore all the short zero derivative areas. The regions R1, R2, R3, and R4 are the real FOG regions which are selected after this process as shown in Figure 8.

#### 3.3.3. FOG False Positive Reduction

After detecting the real FOG-ROIs, we must remove false positives areas. False positives (FP) can happen because of two reasons listed below:

- When a subject is in resting position or standing mode for a while during the experiment causing the foot to have a constant position. This FP reduction is called *Standing Position Removal* step.
- When we have a small movement in the middle of the FOG episode for which the system detects 2 FOGs instead of one FOG. This FP reduction is called *FOG merging* step.

The proposed Kin-FOG follows these two FP removal steps as explained in the next subsections.

##### **Standing** **Position** **Removal**

The Kin-FOG system distinguishes the FOG episodes and the episodes in which a subject is just standing or resting in this phase. The similarity between both is that the foot has almost constant *X* position.

**Figure 9:**
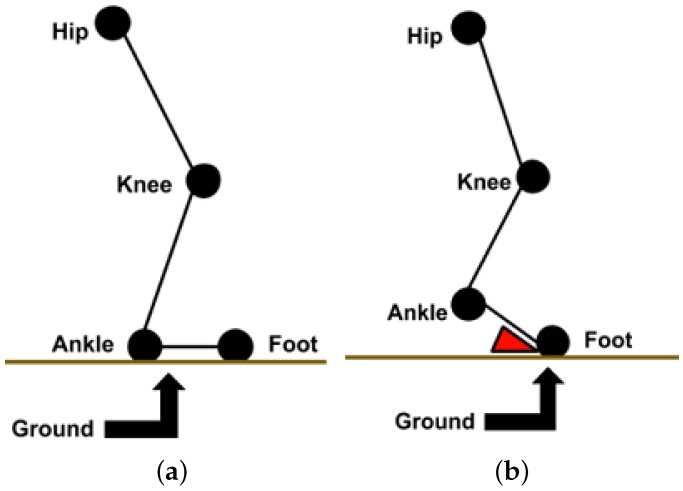
The leg’s joints position: (**a**) standing mode, (**b**) FOG mode.

The leg joints, and hip, knee, ankle, and foot positions, are presented in Figure 9 in FOG and standing mode. Once subjects have FOG the angle between the ankle and foot joints and the ground will be changing; but when they are in the standing mode this angle is stable. Furthermore, the corresponding angle between the foot and ground is shown in Figure 9 with the red triangle. Observe that the angle is almost zero for the standing mode, but it changes in the FOG state. This angle is called *α_AG_* for simplicity.

**Figure 10:**
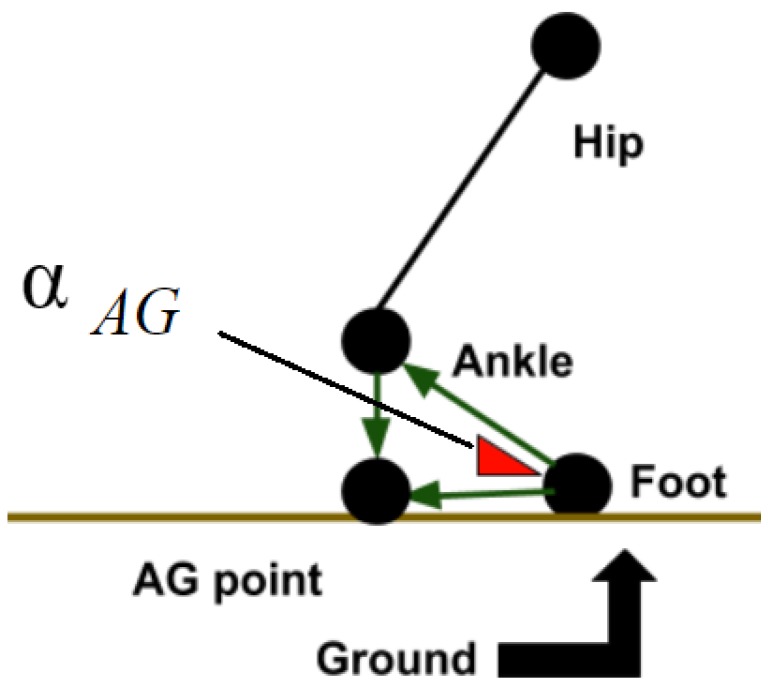
The angle between the ankle-foot joints and the foot-AG point is *α_AG_*.

The *α_AG_* is between two vectors in which the first one (*v*1) is between the ankle and foot joint and the second one (*v*2) is between the foot joint and the point that is the projection of the ankle joint on the ground line (*AG point*). The angle and the vectors are shown in Figure 10. *α_AG_* can be computed by these two vectors based on Equation (3) for each frame which results in an angle list at the end (*AFG* − *list*). AFG stands for ‘Ankle’, ‘Foot’ and ‘Ground.’ The length of the *AFG* − *list* is *n* which is the total number of frames.

(3) $$\alpha_{AG}=\arccos\frac{v1.v2}{∥v1∥∥v2∥}\overset{}{\to }\mathrm{AFG}-\mathrm{list}=\left\{\alpha_{AG1},\alpha_{AG2},\ldots ,\alpha_{AGn}\right\}$$

**Figure 11:**
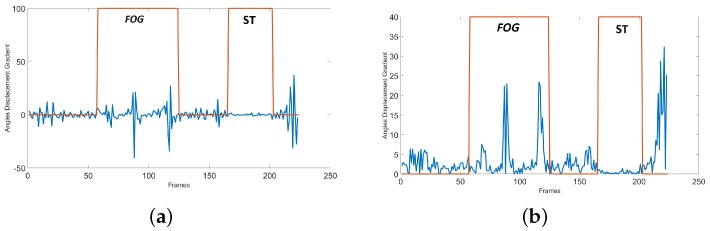
The standing position removal plots for the SW experiment with 1 FOG and 1 ST mode: (**a**) angle displacement plot (*Disp_AFG_*), (**b**) gradient angle displacement plot (*GDisp_AFG_*). ST shows the standing mode.

After computing *α_AG_* for all the frames, the detected FOG episodes from the previous step are assessed in this angle data to remove the standing mode areas. The displacement of the angle list (*AFG* − *list*) is taken to find out the amount of angle change over time for the current subject. The displacement can be computed by Equation (4).

(4) $$Disp_{AFGi}=\alpha_{AGi}-\alpha_{AG(i-1)}\;i\in \left[1:n\right]$$

where *n* is the total number of frames. *α_AGi_* is the angle in frame *i* and *α_AGi_*_−1_ is the angle in frame *i* − 1. Figure 11a shows the current angle displacement in the SW experiment. The FOG episodes show higher changes compared to the standing episodes. The process of finding the resting mode regions is based on the peak values in that area. For finding the amount of change in this signal we get the gradient of the angle displacement data (*Disp* − *AFG*) using Equation (1). The gradient displacement signal is called (*GDisp* − *AFG*). Figure 11b shows the gradient of the current angle displacement. After detecting the FOG episodes (*FOG_Candidates_*), they are evaluated in the (*GDisp_AFG_*) signal. It can be seen that in Figure 11b the FOG regions have peaks with higher values compared to the resting area peak values. Therefore, the process of resting mode removal is based on finding the number of high-value peaks in the FOG candidate regions. The area with some high-value peaks is related to FOG and the area without the high-value peaks shows the resting mode. If the list of peaks in the current episode are [*p*_1_, *p*_2_, …, *p_k_*] where *k* is the total number of peaks, the value for these peaks will be [*GDisp_AFG_*(*p*_1_), *GDisp_AFG_*(*p*_2_), …, *GDisp_AFG_*(*p_k_*)]. After this the number of peaks with value higher than a simple threshold (*PksVal_Thr_*) are computed, which is called *curFOG_PksNum_*. The low number of high-value peaks results in a standing mode which needs to be removed from the FOG candidates list. The removal of resting area is done by making them zero since the FOG area will remain one in the FOG candidate list. The threshold for comparing the number of high-value peaks is *PksNum_Thr_*. The pseudo-code for rest removal is shown in Algorithm 1.

**Algorithm 1** Rest Removal Algorithm
1:
**procedure** Rest Removal
2:    *AFG-list* ← List of angles *α_AG_*.3:    *Disp_AFG_* ← Displacement of angle list *α_AG_*.4:    *GDisp_AFG_* ← Gradient of the *Disp_AFG_*.5:    *FOG_Indxs_* ← *FOG_Candidates_* start and stop points.6:    **for** *ii*,*jj* in *FOG_Indxs_* **do**7:        *curFOG_PksVal_* ← FindPeaks(*GDisp_AFG_*(ii to jj)).8:        *curFOG_PksNum_* ← Length of the *curFOG_PksVal_* > *PksVal_Thr_*.9:        **if** *curFOG_PksNum_* < *PksNum_Thr_* **then**10:           *FOG_Candidates_*(ii to jj) ← 011:        **end if**12:    **end for**13:
**end procedure**



**Figure 12:**
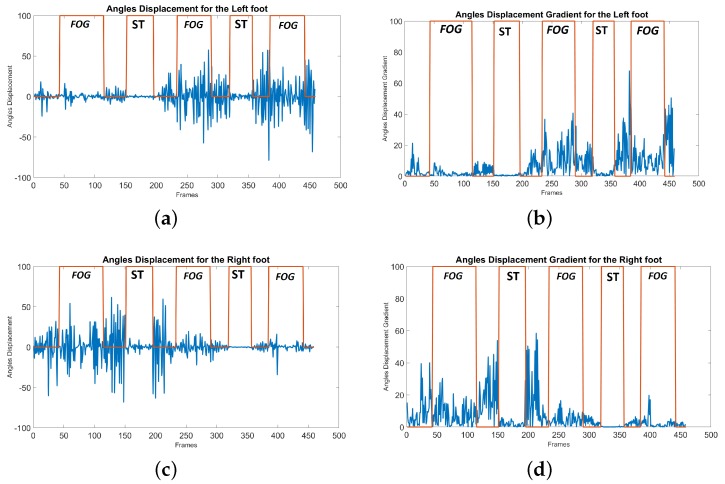
The standing position removal plots for the WWT experiment with 3 FOGs and 2 ST modes: (**a**) angle displacement plot for the left foot (*Disp_AFG_*), (**b**) gradient angle displacement plot for the left foot (*GDisp_AFG_*), (**c**) angle displacement plot for the right foot (*Disp_AFG_*), (**d**) gradient angle displacement plot for the right foot (*GDisp_AFG_*). ST shows the standing mode.

In the SW experiment, only the left foot analysis would be enough to find the standing position and remove FP. However, in the WWT experiment as explained before (Figure 6) the left foot is going to be used for the analysis before turning and the right foot will be used for the analysis after turning. The angle displacement plot (*Disp_AFG_*) for the left and right feet are shown in Figure 12a,c, respectively. Furthermore, the gradient angle displacement plot (*GDisp_AFG_*) for the left and right feet are presented in Figure 12b,d, respectively. The FOG area and the standing mode area (ST) are determined in all the plots.

##### **FOG** **Merging**

**Figure 13:**
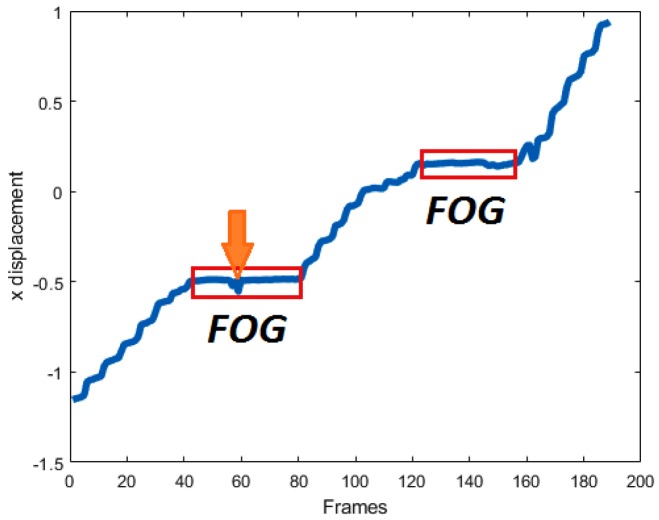
The *X* trajectory plot of a subject with 2 FOGs in the SW experiment. The arrow shows the part which may cause a false positive.

The proposed system finds adjacent FOGs with a small non-FOG area between them and combines these regions into one FOG to decrease the false positive rate of the proposed Kin-FOG system. Figure 13 shows an example of this. The plot is for the SW experiment with 2 FOGs which are marked with labels. Observe that for the first FOG we have a small displacement (marked with the orange array) which causes the system to consider this as two FOGs and eventually report 3 FOGs instead of 2. This part of the system is for getting the right number of FOGs and their corresponding lengths which helps doctors have accurate information about their patients.

**Figure 14:**
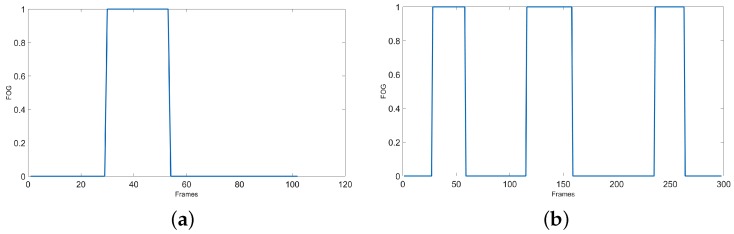
The FOG plots for the subject with the trajectory in Figure 7 for: (**a**) the SW experiment with 1 FOG, (**b**) the WWT experiment with 3 FOGs.

After finding the real FOG-ROIs, the time slot for each of them must be computed. Figure 14 presents the resulting FOG plots for the SW and WWT experiments of the previous signals (Figure 7).

The number of FOGs and their lengths will be computed as well. The number of FOGs is the number of peaks in the FOG plot and the length of FOGs is computed based on Equation (5) for the final detected FOG-ROIs. *FOGLen_fr_* is the length of that particular FOG with the start frame ID (*str_FID_*) and stop frame ID (*sto_FID_*).

(5) $$FOGLen_{fr}=sto_{FID}-str_{FID}$$

The computed length is based on the frame number but we can convert it back to the time space. Since Kinect captures data at 25 fps, the computed FOG length (*FOGLen_fr_*) can be converted to a real-time space (*FOGLen_tr_*) by Equation (6). The computed time unit is in millisecond (ms).

(6) $$FOGLen_{tr}=\frac{FOGLen_{fr}}{25}$$

In summary, the Kin-FOG system uses the foot and ankle joints motion data for FOG assessment for PD patients and outputs the detected FOGs, their lengths, and the time interval when they occurred.

## 4. Experimental Results and Discussions

The Kinect sensor is set up and subjects are asked to do two groups of experiments without standing mode (WOST) and with standing mode (WST). In each of these experiments they perform two types of tasks including SW and WWT. The proposed Kin-FOG system uses the gradient displacement of the foot joint trajectory for finding the list of FOG candidates. It also uses the gradient displacement of the angle between the ankle and foot joints and the ground for false positive reduction from resting mode which has properties similar to FOG modes. Moreover, the merging algorithm at the end ensures that the system reports the correct number of FOGs to the users (doctors, specialists). The proposed system has three initial thresholds, which are *FOGlen_Thr_*, *PKsVal_Thr_* and *PKsNum_Thr_*. The values for these thresholds are *FOGlen_Thr_* = 10, *PKsVal_Thr_* = 5 and *PKsNum_Thr_* = 3 which are constant for all the subjects in the SW and WWT experiments. Experimental results for each group are illustrated below.

The ground truth label for each subject is obtained based on the video data captured by Kinect. The video data is assessed and the label for each frame is determined. The ground truth label has three types of values consisting of *non-related*, *FOG* and *non-FOG*. The *non-related* frames are the ones that are not related to the experiments which are before point *A* and after point *B* (Figure 4). These frames are ignored for the evaluation part. The *FOG* frames are the ones for which a subject has FOG during the time interval. The rest of the frames are labeled as *non-FOG*. The evaluation of the proposed Kin-FOG frame is based on the FOG detection part for which quantitative results will be provided. After that, the number and length of the prediction will be evaluated by comparing to the real number and length of the FOG episodes. Other than quantitative results, qualitative assessment is also reported by the proposed FOG assessment system; such as the plots of FOG episodes for the subjects.

### 4.1. WOST Experiment

**Figure 15:**
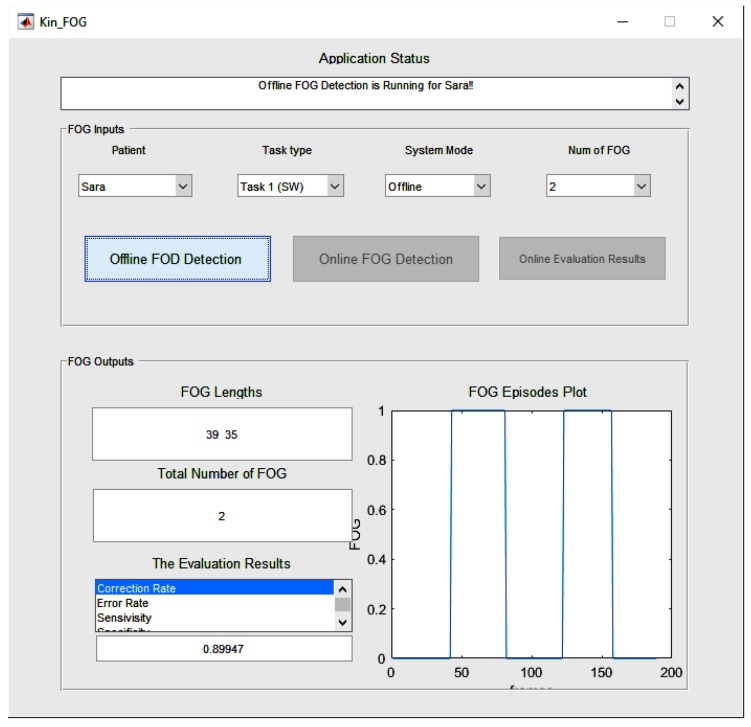
The main GUI of the proposed Kin-FOG system for FOG assessment of PD patients.

Video data is collected for each subject in WOST for different numbers of FOGs. As mentioned before for SW, the subjects perform 1 and 2 FOGs and for WWT, 3, 4, and 5 FOGs. The FOG episodes happen randomly for each subject. The reason behind performing different numbers of FOGs in each experiment is having a more reliable and general system which works in different situations. The graphical user interface (GUI) is designed for our proposed Kin-FOG system, as shown in Figure 15. The proposed system has two modes including: (1) Offline FOG assessment, (2) Online FOG assessment, which both give useful FOG information on PD patients to the neurologists and care providers. The offline part gives all the required FOG information for the entire data captured by Kinect for the subjects. However, the online part is for visualization of the FOG status for each frame separately. The main GUI has two sections which are *’FOG Inputs’* (the inputs of the system) and *’FOG Output’* (the outputs of the system). The *’FOG Inputs’* part has options for choosing the patient, the task type (SW, WWT), the system’s mode (offline, online) and the number of FOGs (for SW:[1,2] and for WWT:[3,4,5]). The *Num of FOG* parameter is for evaluation purpose since we need to get the right ground truth labels based on the selected criteria.

The offline FOG assessment uses the proposed FOG detection method and reports the *total number of FOGs*, the *FOG lengths*, and *FOG episodes plot*. The proposed system not only reports quantitative results but also provides qualitative information, such as plots of FOG episodes. This information is useful for specialists to see in what situations patients have more FOGs. A patient might have a problem just when they turn but not when they walk straight. Therefore, they need to have treatment just for that special situation which cause FOG for them. This way doctors can provide the most efficient treatment, such as cueing to their patients. The FOG episodes plot illustrates the FOG time slots including the start frame IDs and the stop frame IDs. Furthermore, the *Evaluation Results* section presents different criteria to evaluate the Kin-FOG system to assess the current subject. These criteria are *correct rate*, *error rate*, *sensitivity* and *specificity*.

**Figure 16:**
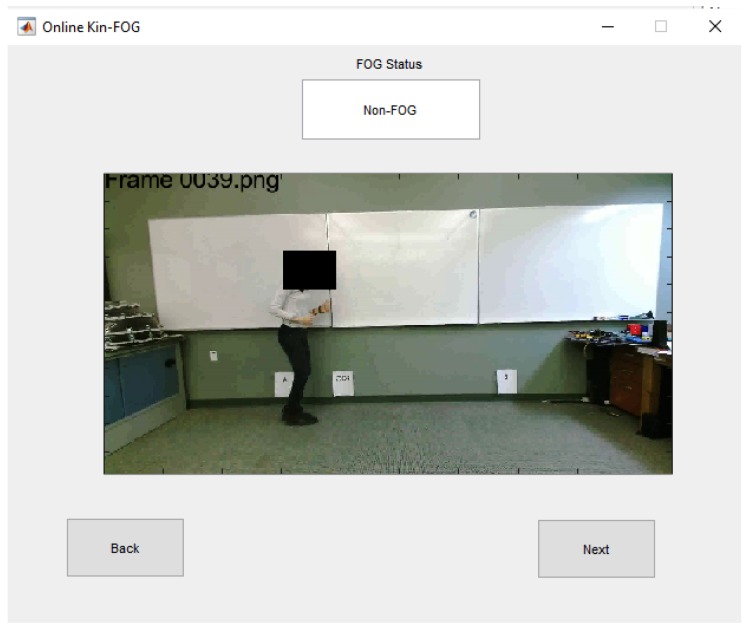
The online Kin-FOG GUI.

The online mode of the system has a separate GUI which is presented in Figure 16. The main GUI gets all the required information from the *FOG Inputs* part and shows the corresponding video frames for the particular subject in the online GUI. The online Kin-FOG presents the FOG status for each frame to the users (specialists or care providers) as shown in Figure 16.

**Figure 17:**
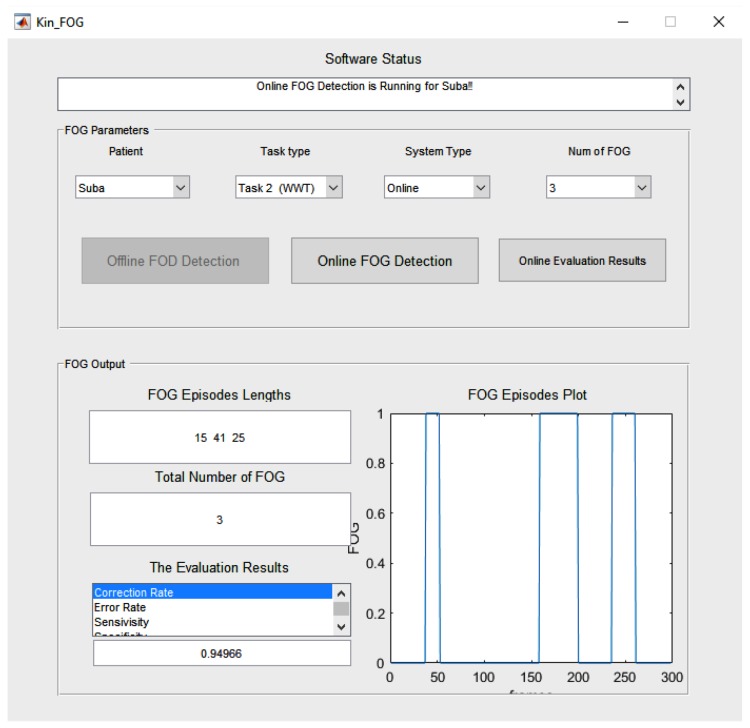
The Kin-FOG output for one of the subjects in the online mode, which consists of number of FOGs, their time slots, and their lengths. In addition, the performance of the Kin-FOG is also presented in the *’Evaluation Results’* section.

In addition, the *’Online Evaluation Result’* button in the main GUI can show the evaluation results for the online mode of the system. Figure 17 shows the results of the online mode of the Kin-FOG for one of the subjects in the WWT experiment with 3 FOGs. The results show 3 FOGs with their lengths and the plot for showing the time slots. Also, the status of the Kin-FOG system is shown on top of the main GUI which presents exactly what the system is doing. For instance, in this case it shows running for the subject with name *’Suba’* and the Kin-FOG system mode is online.

Quantitative results are shown for the proposed Kin-FOG system in two forms including: (1) local (subject-dependent), and (2) general (subject-independent) similar to the evaluation in [8]. Table 3 shows the numerical evaluation results for different subjects using the above-mentioned criteria. The local evaluation for each subject is reported and the general evaluation is presented in the last row of the table. As the results show, the proposed Kin-FOG system has high accuracy in both experiments (SW and WWT).

The predicted number of FOGs and their lengths for each subject are presented in Table 4. *’PN’* means predicted FOG number and *’PL’* means predicted FOG lengths for each subject. The predicted number of FOGs are correct in all cases except 3 which are encircled in Table 4. The general evaluation is done for FOG number prediction in this part. Since we have 5 subjects and the number of evaluation tasks for each of them is 5 (2 FOGs types for the SW and 3 FOGs types for the WWT), the total number of experimental cases is 25. The general accuracy of the proposed Kin-FOG system in FOG number prediction can be computed based on Equation (7).

(7) $$PN_{accuracy}=\left(\left(25-3\right)/25\right)*100=88$$

The local accuracy for predicting the number of FOGs for each individual subject is listed in Table 4, last column. Each subject has 5 experimental cases (SW-FOG 1 and 2, WWT-FOG 3, 4, and 5). Thus, based on the number of correct predictions for FOG numbers in different cases the local accuracy for each subject is computed. In addition, FOG detection and FOG number prediction gives the corresponding FOG lengths. The predicted lengths are also presented in Table 4. The predicted lengths for the FOGs for all the subjects are compared with the real FOG lengths in each task for different experiments. The difference between the predicted FOG lengths and the real FOG lengths in most cases is less than 5 frames.

### 4.2. WST Experiment

Video data is collected for each subject in WST for SW and WWT tasks. In SW, the subjects perform 1 FOG and 1 standing mode and for WWT, they perform 3 FOGs and 2 standing modes. The FOG and standing time slots are random for different subjects.

In this part of our research, we evaluate our system’s ability to distinguish between the FOG and standing modes. The same GUIs are used for the FOG assessment in WST experiment for showing the detected FOGs, their lengths, and their plots. Table 5 presents the ability of Kin-FOG for detecting the real FOG episodes when we have the standing mode besides the FOG modes in our captured data with Kinect. The local evaluation for each subject is reported beside the general evaluation in the last row of Table 5.

Our FOG assessment system is simpler than the proposed gait assessment system for stroke patients [11] and the one proposed for equestrian sport [33]; since it works based only on one individual sensor which provides the motion and video data together while others use fusion of data from different sensors. Furthermore, since the sensor is non-wearable, it is more comfortable for PD patients who are mostly elderly. Kinect provides video data besides the motion data; but, in the proposed gait analysis system by [11,33], the authors use other optical sensors or cameras for collecting the video data in order to evaluate their proposed method at the end. Self-administration of Kinect compared to wearable sensors is simpler making it easier to set up. However, the proposed gait assessment system in [11] claimed that they solved the sensor drift problem by zero velocity update algorithm to address the challenge of using wearable sensors in an out-of-lab environment. It needs to be mentioned that the proposed system in [11,33] is applicable in outdoor environments but our FOG assessment system only works inside a lab, a clinic or a patient’s home. The proposed Kin-FOG system is a useful tool for the neurologists and others who need to get the FOG information about patients.

Our proposed FOG assessment system is evaluated for healthy subjects performing simulations; however, in future work, it will be evaluated for real PD patients. The proposed foot-off detection algorithm by Amini et al. [31] was first evaluated for healthy subjects in [35] and later on PD patients in [31]. We have the same plan for our research. Even though the number of subjects in our experiments is limited, it needs to be noted that research with few subjects can be faster for enlisting subjects, looking into their records, or performing biochemical investigations [36].

## 5. Conclusions

FOG is one of the major motor symptoms of PD patients which can cause falls. Since, PD patients are mostly elderly, falling results in fractures and even death. Thus, it is critical to find a way to detect FOG and stop patients from falling. Neurologists require accurate information about the FOG status of patients for giving proper and effective treatments. In this research, an automatic, accurate and fast FOG assessment application (Kin-FOG) is designed, which works based on the video and motion data captured by a Microsoft Kinect sensor. The gradient of the displacement for foot joint trajectory is used for gait analysis and distinguishing between the FOG episodes. On the other hand, the angle between the foot and the ground is used for false positive reduction resulting from having resting modes in our gait analysis. Evaluation of the proposed method is done based on two types of experiments with and without resting modes (WST and WOST) and different modes (SW, WWT). Moreover, different number of FOGs are performed by the subjects to have a more reliable and general result of our assessment system. The outputs of the Kin-FOG system are the numbers and lengths of FOGs and the time interval for each of them. Kin-FOG is evaluated using data captured for 5 healthy subjects who are trained to imitate FOG. The experimental results show the local (user-dependent) and general (user-independent) evaluation for all the experiments. The overall accuracy rate for FOG prediction is around 90% for both experiments which demonstrates the ability of the proposed system in FOG assessment. The proposed Kin-FOG system is low-cost, accurate, and easy to use for FOG assessment of PD patients. In addition, the proposed system can be used remotely at patients’ homes for sending the FOG status to doctors. Even though the Kin-FOG is evaluated for a small data set of healthy subjects performing simulations, the ability of the novel FOG assessment system will be evaluated for a larger number of real PD patients in our future work. 

## Figures and Tables

**Table 1:** Subject demographics.

*Subject Characteristics*	*Range*	*Standard Deviation*
***Age***	26–33	1.87
***Height (cm)***	170–186	5.04
***Weight (Kg)***	55–85	9.09
***BMI***	17.8–24.8	2.44

**Table 2:** The number of FOGs in each experiment and different modes.

Experiments/Modes	SW	WWT
**WOST**	1,2	3,4,5
**WST**	1	3

**Table 3:** Quantitative results of the Proposed Kin-FOG system in the WOST experiment for PD patients. The local (subject-dependent) and the general (subject-independent) results are reported.

	*SW*				*WWT*			
***Subjects\Evaluation***	***Correction Rate***	***Error Rate***	***Sensitivity***	***Specificity***	***Correct Rate***	***Error Rate***	***Sensitivity***	***Specificity***
***Subject 1***	0.9525	0.0475	0.9268	0.9912	0.9387	0.06130	0.9112	0.9931
***Subject 2***	0.7979	0.2021	0.4726	1	0.9608	0.0392	0.9545	0.9611
***Subject 3***	0.9503	0.0497	0.9342	1	0.7896	0.2104	0.8499	0.6128
***Subject 4***	0.9360	0.064	0.9651	0.8919	0.8850	0.1150	0.8580	0.9644
***Subject 5***	0.9618	0.0382	0.9725	0.9306	0.9132	0.0868	0.8946	0.9755
***General Evaluation***	0.9197	0.08030	0.8542	0.9627	0.8960	0.1040	0.8910	0.9014

**Table 4:** The predicted FOG numbers and lengths by the proposed Kin-FOG system in different tasks with different number of FOGs. *PN* is the predicted number of FOGs and *PL* is the predicted lengths of FOGs.

*Task Type*	*SW*				*WWT*						*Evaluation*
***Task FOG Number***	***FOG 1***		***FOG2***		***FOG3***		***FOG4***		***FOG5***		***Accuracy***
***Prediction Number and Length of FOGs***	***PN***	***PL***	***PN***	***PL***	***PN***	***PL***	***PN***	***PL***	***PN***	***PL***	***PN***
***Subject 1***	1	“60”	2	“54,35”	④	“35,17,46,37”	4	“41,46,42,35”	5	“58,35,10,34,45”	80
***Subject 2***	1	“44”	2	“40,50”	3	“46,52,28”	4	“42,33,40,46”	5	“39,37,38,43,38”	100
***Subject 3***	1	“24”	2	“28,42”	3	“25,21,20”	4	“31,25,21,20”	⑥	“23,14,10,18,13,16”	80
***Subject 4***	1	“22”	2	“39,35”	3	“21,37,22”	4	“35,19,28,29”	5	“13,24,39,26,31”	100
***Subject 5***	1	“18”	2	“29,19”	3	“15,41,25”	⑤	“16,18,19,19,15”	5	“22,20,41,19,16”	80

**Table 5:** The WST experiment’s quantitative results for the Proposed Kin-FOG system in FOG assessment of PD patients. The local (subject-dependent) and the general (subject-independent) results are reported.

	*SW*				*WWT*			
***Subjects\Evaluation***	***Correction Rate***	***Error Rate***	***Sensitivity***	***Specificity***	***Correction Rate***	***Error Rate***	***Sensitivity***	***Specificity***
***Subject 1***	0.7640	0.2360	0.5581	1	0.8693	0.1307	0.8037	0.9913
***Subject 2***	0.9063	0.0938	0.8981	0.9254	0.8584	0.1416	0.9672	0.6973
***Subject 3***	0.9713	0.0287	0.9597	1	0.8610	0.1390	0.9174	0.6964
***Subject 4***	0.9792	0.0208	0.9664	1	0.9462	0.0538	0.9072	1
***Subject 5***	0.9628	0.0372	0.9479	1	0.9767	0.0233	0.9680	0.9946
***General Evaluation***	0.9167	0.0833	0.8661	0.9851	0.9023	0.0977	0.9127	0.8759
